# Supplementary material for: Statistical analysis of the impact of environmental temperature on the exponential growth rate of cases infected by COVID-19
Source: PLoS One. 2020 May 29;15(5):e0233875. doi: 10.1371/journal.pone.0233875 (PMC7259789; doi:10.1371/journal.pone.0233875)
Supplement: Attachment — Submitted filename: Response to referees.pdf [file pone.0233875.s002.pdf]

ID: PONE-D-20-11474

Title: Statistical analysis of the impact of environmental temperature on the exponential growth rate of cases infected by COVID-19

PLOS ONE

Author: G. Livadiotis

Response to Referees

We would like to thank both the referees for their good words and recommendations for publication of the paper. We are also grateful for their comments, which have all of them been addressed and helped for improving the impact of the paper.

Together with this response, we have included a "track changes" version as well as a clean version of the revised manuscript. In the "track changes" version we mark all the changes-corrections in regards to all referees' comments. Throughout this reply, the original referees' comments are reproduced verbatim in blue with our general comments on each point following in black text.

Referee #1:

1. Theory Section, Equation 1.: I recommend that you have the relevant citations before introducing the Equation.

   This is a basic model produced by this paper. We have made this clearer.

2. Theory Section, 4th paragraph: In the specific sentence I believe the main point is shown by both panels in Figure 3. Wouldn't also a semi-plot log make this point even more clear?

   We have included a semi-log input panel. We also improve the scale numbering of this figure. In addition, we replot Figure 2(a).

3. Theory Section, after Equation 5, λ depends on Ro, which could potentially depend on social activities. On the other hand, 4th paragraph of introduction states that the measures do not affect the exponential rate, indicating that there is no potential dependence of parameter λ on Ro. In other words, by reading the introduction I would expect that measures appear only in the negative feedback factor *I*, and not in the *E* factor of model in equation (1). I think this point can get a bit clearer either in the introduction or in the theory because it seems contradictive from the first read.

   It should be pointed out that the *culture*, that is, the features of social activities of a certain population, and *measures*, are different concepts and thus, are represented by different modeling factors. Effective measures can cease the social activities, but they do not alter the culture characteristics of these activities; once measures are risen, the social activities will be back again and characterized by the same culture features.

   In terms of modeling, measures appear only in the negative feedback factor *I*, and not in the *E* factor of model in Eq.(1). On the other hand, the culture, together with temperature, are the two main parameters affecting R_0, and thus λ. In the linear relationship of λ with temperature, the slope appears to be universal quantity, while the intercept depends on the culture of the examined population.

   We have corrected the revised version of the text to make the above notes clear.

4. Methodology section, point ii): How does the author detects the time interval corresponding to the exponential growth?
   The exact limits of the linear relationship (on semi-log scale of λ) are taken for those where the goodness of linear fitting is maximized.

5. Results section, paragraph 4: There is a typo in the second to last sentence (“Tthe” should be “The”)
   Corrected. We have also double-checked the paper and corrected all other typos.

Referee #2:

There are several awkward expressions and misusage of certain English words. Here are few of them.

1. The statement in line 6 in the abstract is in contradiction of the last sentence and should be corrected: (there is a positive correlation between the average temperature and ...)
Explained.

2. In the introduction, the word "decay" should be replaced by "decline". In line 5, the word outburst is used. as a verb which it is not and should be replaced by "explode".
Typically in literature, the population is said to be declined; the term outburst has been also corrected accordingly.

3. line 5 in Section 2.2, will spread the disease to over: should read will spread the disease over.
Corrected.

3. In section 2.1, The differential equation is written in a non-traditional way x^._t instead of x^.(t). The subscripts are usually reserved for difference equations. The author. needs to explain clearly how he came up with this simple model and describe clearly the functions E and I.
Corrected.

4. The author needs t o explain better the relationship between lambda and R_0 and how he got the equations N_(t+1)=R_0 N_t
This and all related equations have now been clearly written and explained.

5. The author needs to give a better explanation of the derivations of equations 6-10. The author needs to elaborate on the criticality of Arrhenius equation in modeling the effect of temperature on the corona virus.
Note that the Arrhenius behavior is just one possible way to explain the temperature dependence, and that is the reason why we examine also fitting of λ against inverse temperature, 1/T. Nevertheless, we examine also the simple linear relationship of λ against T, even though is not Arrhenius connected relationship.
The above risen issue, as well as Eqs.(6-10), and all related equations have now been clearly written and explained.

Again, we are thankful for reviewing this important work.

George Livadiotis
