# Supplementary material for: Statistical analysis of the impact of environmental temperature on the exponential growth rate of cases infected by COVID-19
Source: PLoS One. 2020 May 29;15(5):e0233875. doi: 10.1371/journal.pone.0233875 (PMC7259789; doi:10.1371/journal.pone.0233875)
Supplement: Attachment — Submitted filename: Review_of_Livadiotis_2020.docx [file pone.0233875.s001.docx]

Dear Editor,

I have read and reviewed the paper “Statistical analysis of the impact of environmental temperature on the exponential growth rate of cases infected by COVID-19” by G. Livadiotis.

The original research of this paper demonstrates the negative correlation between the exponential growth rate of the cases infected by COVID-19 for US and Italian regions and the environmental temperature.

The author uses valid statistical methods to establish an accurate anti-correlation of the infected cases and the environmental temperature. Numerous statistical tests validate the results.

The study derives the critical temperature which may eliminate the daily infected cases. This is a purely statistical approach to such an important matter, and with extra caution, the results could be very useful for future decisions regarding measures against the pandemic.

I therefore recommend the publication of the manuscript after the minor changes I list below:

1. Theory Section, Equation 1.: I recommend that you have the relevant citations before introducing the Equation.
2. Theory Section, 4^th^ paragraph: In the specific sentence I believe the main point is shown by both panels in Figure 3. Wouldn’t also a semi-plot log make this point even more clear?
3. Theory Section, after Equation 5, λ depends on Ro, which could potentially depend on social activities. On the other hand, 4^th^ paragraph of introduction states that the measures do not affect the exponential rate, indicating that there is no potential dependence of parameter λ on Ro. In other words, by reading the introduction I would expect that measures appear only in the negative feedback factor *I*, and not in the *E* factor of model in equation (1). I think this point can get a bit clearer either in the introduction or in the theory because it seems contradictive from the first read.
4. Methodology section, point ii): How does the author detects the time interval corresponding to the exponential growth?
5. Results section, paragraph 4: There is a typo in the second to last sentence (“Tthe” should be “The”)
